# Digital Health Technology Interventions for Improving Medication Safety: Systematic Review of Economic Evaluations

**DOI:** 10.2196/65546

**Published:** 2025-02-05

**Authors:** Widya Norma Insani, Neily Zakiyah, Irma Melyani Puspitasari, Muhammad Yorga Permana, Kankan Parmikanti, Endang Rusyaman, Auliya Abdurrohim Suwantika

**Affiliations:** 1 Department of Pharmacology and Clinical Pharmacy Universitas Padjadjaran Sumedang Indonesia; 2 Centre of Excellence for Pharmaceutical Care Innovation Universitas Padjadjaran Sumedang Indonesia; 3 School of Business & Management Bandung Institute of Technology Bandung Indonesia; 4 Department of Mathematics Universitas Padjadjaran Sumedang Indonesia

**Keywords:** digital health technology, drug safety, adverse drug events, medication errors, patient safety

## Abstract

**Background:**

Medication-related harm, including adverse drug events (ADEs) and medication errors, represents a significant iatrogenic burden in clinical care. Digital health technology (DHT) interventions can significantly enhance medication safety outcomes. Although the clinical effectiveness of DHT for medication safety has been relatively well studied, much less is known about the cost-effectiveness of these interventions.

**Objective:**

This study aimed to systematically review the economic impact of DHT interventions on medication safety and examine methodological challenges to inform future research directions.

**Methods:**

A systematic search was conducted across 3 major electronic databases (ie, PubMed, Scopus, and EBSCOhost). The PRISMA (Preferred Reporting Items for Systematic Reviews and Meta-Analyses) guidelines were followed for this systematic review. Two independent investigators conducted a full-text review after screening preliminary titles and abstracts. We adopted recommendations from the Panel on Cost-Effectiveness in Health and Medicine for data extraction. A narrative analysis was conducted to synthesize clinical and economic outcomes. The quality of reporting for the included studies was assessed using the CHEERS (Consolidated Health Economic Evaluation Reporting Standards) guidelines.

**Results:**

We included 13 studies that assessed the cost-effectiveness (n=9, 69.2%), cost-benefit (n=3, 23.1%), and cost-utility (n=1, 7.7%) of DHT for medication safety. Of the included studies, more than half (n=7, 53.9%) evaluated a clinical decision support system (CDSS)/computerized provider order entry (CPOE), 4 (30.8%) examined automated medication-dispensing systems, and 2 (15.4%) focused on pharmacist-led outreach programs targeting health care professionals. In 12 (92.3% ) studies, DHT was either cost-effective or cost beneficial compared to standard care. On average, DHT interventions reduced ADEs by 37.12% (range 8.2%-66.5%) and medication errors by 54.38% (range 24%-83%). The key drivers of cost-effectiveness included reductions in outcomes, the proportion of errors resulting in ADEs, and implementation costs. Despite a significant upfront cost, DHT showed a return on investment within 3-4.25 years due to lower cost related with ADE treatment and improved workflow efficiency. In terms of reporting quality, the studies were classified as good (n=10, 76.9%) and moderate (n=3, 23.1%). Key methodological challenges included short follow-up periods, the absence of alert compliance tracking, the lack of ADE and error severity categorization, and omission of indirect costs.

**Conclusions:**

DHT interventions are economically viable to improve medication safety, with a substantial reduction in ADEs and medication errors. Future studies should prioritize incorporating alert compliance tracking, ADE and error severity classification, and evaluation of indirect costs, thereby increasing clinical benefits and economic viability.

## Introduction

Medication-related harm, including adverse drug events (ADEs) and medication errors, represents a significant burden in clinical care [1]. A previous systematic review showed that 3% of patients in various health care settings experience preventable medication-related harm worldwide, with a quarter classified as severe or potentially life threatening [2]. Unsafe medication practices can occur at various stages of the medication process, including prescribing errors, where nearly 50% of medication errors occur (eg, inappropriate drugs for age and condition, incorrect dosage, contraindication, and drug-drug interactions (DDIs) overlooked) [3,4]; dispensing errors; administration errors; and inadequate monitoring (eg, hypokalemia due to inadequate renal and electrolyte monitoring among diuretic users) [5,6]. Additional costs associated with medication errors have been estimated at US $42 billion annually, indicating a significant burden of unsafe medication practices for patients and the health system [7].

Digital health technology (DHT) applies information and communication technology to enhance health care outcomes [8]. DHT interventions range from a clinical decision support system (CDSS)/computerized provider order entry (CPOE) or electronic prescribing, automated medication-dispensing systems, telemedicine, and mobile health (mHealth) apps to telephone or text message reminders [9-12]. DHT may assist in supporting clinical decisions and enhancing the monitoring of medication use [13,14]. Several studies have shown that DHT may confer benefits by reducing ADEs and medication errors in both hospital and community settings [15-17]. Previous systematic reviews have examined the clinical impact of DHT interventions (ie, CPOE) to improve medication safety [18,19], but none have examined the economic impact of such interventions.

Given the increasing application of DHT, there is an urgent need to determine the economic benefits of DHT to allow clinicians to decide whether to implement DHT strategies [20]. Although DHT interventions can potentially improve clinical outcomes, their additional expense for infrastructure, implementation, and maintenance may hinder their adoption compared to standard care [21,22]. This study aimed to systematically review the economic impact of DHT interventions on medication safety and examine the methodological challenges in these interventions. Such understanding can better inform resource allocation and policy decisions for strengthening health system capacity [23].

## Methods

### Search Strategy

This systematic review followed the PRISMA (Preferred Reporting Items for Systematic Reviews and Meta-Analysis) guideline. A systematic search was performed across 3 major electronic databases (PubMed, Scopus, and EBSCOhost) to identify studies investigating economic evaluations of DHT to improve medication safety outcomes (ie, reduction in ADEs and medication errors). The search strategies included 3 categories of terms related to:

- DHT interventions, defined as strategies that use information and communication technology to enhance health outcomes (ie, reduction in ADEs and medication errors). To ensure comprehensive coverage of all available evidence, we included a broad range of DHT interventions, including a CDSS/CPOE, electronic prescribing, telemedicine, telepharmacy, automated medication-dispensing systems, mHealth apps, and telephone or text message reminders. Comparators included paper-based prescribing, traditional floor stock storage, and standard pharmaceutical care.
- Medication safety outcomes (ie, changes in the ADE and medication error rate). An ADE is defined as “an injury resulting from the use of a drug,” which includes harm resulting from either errors or medication-inherent effects [24]. The term “medication error” refers to “any error in the process of prescribing, dispensing, or administering a drug, which may or may not result in harm” [25]. A previous study showed that although medication errors are common, only around 1% result in actual harm or ADEs, which might be because these errors have little potential for injury or they are intercepted before an adverse outcome occurs [24,26].
- Economic evaluations (ie, cost-benefit analysis [CBA], cost-effectiveness analysis [CEA], cost-utility analysis [CUA], and cost minimization analysis [CMA]).

Full details of the search strategy are provided in Table 1. An additional search of reference lists of relevant reviews and included studies was performed.

**Table 1:** Search strategy of DHT^a^ interventions for medication safety.

Type of key terms	Search strategy
Terms related to DHT interventions	Telemedicine[MeSH^b^] OR telepharmacy[tiab] OR computerized provider order entry[tiab] OR computerized physician order entry[tiab] OR computer provider entry[tiab] OR clinical decision support[tiab] OR automated medication system[tiab] OR automated pharmacy system[tiab] OR bar coding[tiab] OR electronic medication order entry[tiab] OR electronic medication management system[tiab] OR electronic prescribing[tiab] OR ePrescribing OR electronic prescription[tiab] OR electronic medication administration records[tiab] OR electronic system*[tiab] OR automated dispensing[tiab] OR computerized reminder system[tiab] OR information technology[tiab] OR medication ordering entry[tiab] OR electronic medication ordering and administration system[tiab] OR remote consultation[MeSH] OR electronic consult*[tiab] OR digital technolog*[tiab] OR teleconsult*[tiab] OR mhealth[tiab] OR m-health[tiab] OR multimedia[tiab] OR virtual[tiab] OR mobile health[tiab] OR telemedicine[tiab] OR electronic health record[tiab] OR telehealth[tiab] OR telecare[tiab] OR telehealth care[tiab] OR mobile health intervention*[tiab] OR mobile applications[tiab] OR mobile telemedicine[tiab] OR mcare[tiab] OR m-care[tiab] OR mobile communication[tiab] OR mobile technolog*[tiab] OR multimedia technolog*[tiab] OR mobile devic*[tiab] OR app[tiab] OR apps[tiab] OR mobile app*[tiab] OR website*[tiab] OR internet consultation*[tiab] OR internet monitoring[tiab] OR video consultation*[tiab] OR video monitoring[tiab] OR telephone*[tiab] OR mobile phone*[tiab] OR smart phone*[tiab] OR smart-phone*[tiab] OR text messag*[tiab] OR text messaging[tiab] OR SMS[tiab] OR short messag*[tiab] OR multimedia messag*[tiab] OR multi-media messag*[tiab] OR website platform[tiab] OR web-based medication platform[tiab] OR web-based application[tiab] OR web-based tool[tiab] OR electronic health[tiab] OR ehealth[tiab] OR e-health[tiab]
Terms related to medication safety outcomes	Drug-related side effects and adverse reactions [MeSH] OR adverse drug reaction*[tiab] OR adverse drug event*[tiab] OR drug related problem*[tiab] OR medication related problem[tiab] OR drug therapy problem*[tiab] OR drug safety[tiab] OR medication safety[tiab] OR medication error*[tiab] OR prescribing error*[tiab] OR prescription error*[tiab] OR dispensing error*[tiab] OR administration error*[tiab] OR inappropriate prescribing[tiab] OR inappropriate medication*[tiab] OR drug complication*[tiab]
Terms related to economic evaluations	Cost and cost analysis[MeSH] OR cost-benefit analysis[MeSH] OR cost-effectiveness[tiab] OR cost utility[tiab] OR cost minimi*[tiab] OR economic evaluation[tiab] OR economic analysis[tiab]

^a^DHT: digital health technology.

^b^MeSH: Medical Subject Headings.

### Inclusion and Exclusion Criteria

We included studies that reported health economic evaluations of DHT to improve medication safety outcomes (ie, ADEs and medication errors) compared to standard care. Detailed information related to inclusion criteria is provided in Table 2. We excluded studies where the intervention did not include DHT, studies that did not report medication safety outcomes, studies without full economic evaluation, non-English studies, conference abstracts, and editorials.

**Table 2:** PICO^a^ framework and inclusion criteria of the study.

Framework item	Inclusion criteria
Population/problem	Patients receiving medication at any point of care, including in hospital and community settings
Intervention	Any DHT^b^ intervention, including: CDSS^c^/CPOE^d^ Automated medication-dispensing system Telepharmacy, telemedicine mHealth^e^ app Other
Comparator	Standard care (eg, paper-based prescribing, traditional floor stock storage)
Outcome	Reduction in ADEs^f^ and medication errors
Study type	A full economic evaluation of DHT to improve medication safety categorized as CBA^g^, CEA^h^, CUA^i^, and CMA^j^

^a^PICO: population/problem, intervention, comparator, outcome.

^b^DHT: digital health technology.

^c^CDSS: clinical decision support system.

^d^CPOE: computerized provider order entry.

^e^mHealth: mobile health.

^f^ADE: adverse drug event.

^g^CBA: cost-benefit analysis.

^h^CEA: cost-effectiveness analysis.

^i^CUA: cost-utility analysis.

^j^CMA: cost minimization analysis.

### Screening and Data Extraction

The database-screening results were exported to the Mendeley reference manager library and examined for duplicates. Two investigators (authors WNI and NZ) independently conducted a full-text review after screening the preliminary titles and abstracts. Any discrepancies between the 2 reviewers were resolved through discussion. We adopted recommendations from the Panel on Cost-Effectiveness in Health and Medicine for data extraction. Data extracted included (1) study characteristics; (2) clinical and economic outcomes, including the incremental cost-effectiveness ratio (ICER), the cost-benefit ratio (CBR), and the return on investment (ROI); (3) cost components; (4) methodological challenges; and (5) quality of reporting. All monetary values were converted to 2024 US dollar values using the Campbell and Cochrane Economics Methods Group–Evidence for Policy & Practice Information Centre Cost Converter [27].

### Quality of Reporting

Reporting quality was assessed using the CHEERS (Consolidated Health Economic Evaluation Reporting Standards) 2022 checklist. The checklist included 28 items, classified into 6 categories: (1) title and abstract, (2) introduction, (3) methods (including choice of model, health outcomes, and measurement of effectiveness), (4) results, (5) discussion, and (6) others. Based on the reporting quality, the included studies were categorized as excellent (score 100%), good (score 75%-99%), moderate (score 50%-74%), and low (score ≤49%) [28].

## Results

### Study Selection

**Figure 1:**
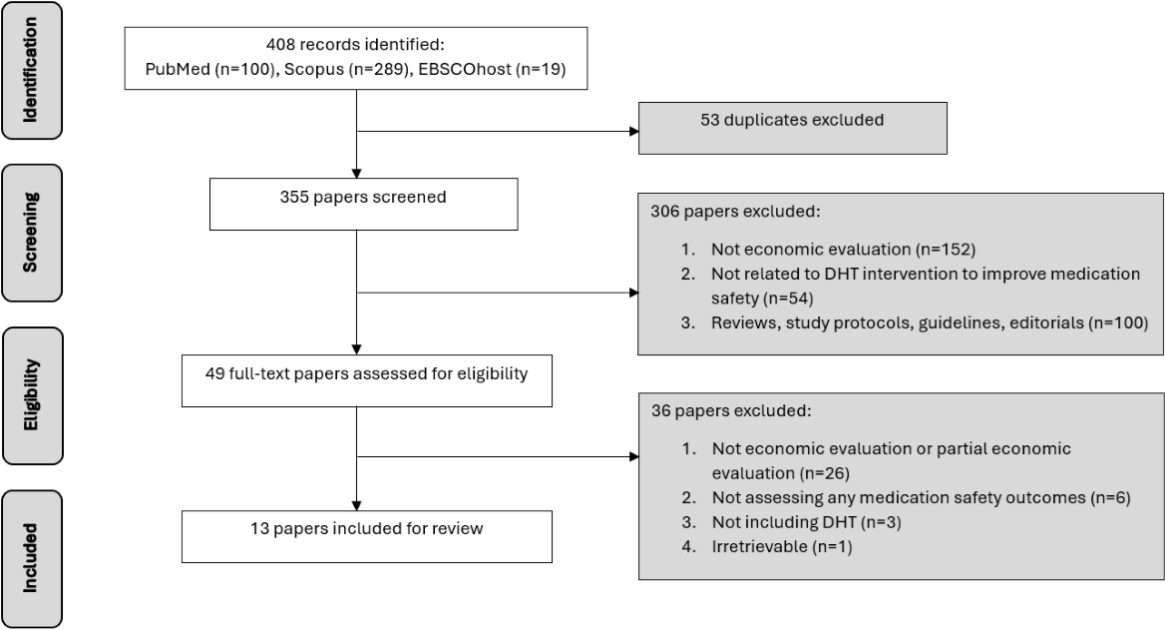
PRISMA flowchart of study selection. PRISMA: Preferred Reporting Items for Systematic Reviews and Meta-Analyses.

A total of 408 citations were retrieved from the electronic databases. After removing duplicates, 355 (87%) papers remained for evaluation based on titles and abstracts, yielding 49 (13.8%) records eligible for full-text assessment. A total of 13 (26.5%) economic evaluation studies [10,12,15,17,29-37] were finally included in this systematic review. Most of the studies included CEA (n=9, 69.2%) [12,15,17,29-34], followed by CBA (n=3, 23.1%) [10,35,36], and CUA (n=1, 7.7%) [37]. The included studies were predominantly conducted in hospital inpatient settings (n=10, 76.9%) [10,12,17,30-36]. The study selection flowchart based on PRISMA guidelines is provided in Figure 1.

### Overview of Key Characteristics

The most frequently implemented DHT intervention was the CDSS/CPOE or electronic medication record system. This intervention typically replaced traditional paper-based prescribing, with a safety alerts feature to assist clinicians in making informed decisions during the prescribing process [12,17,29,30,33-35]. A wide range of features were used in the included studies, ranging from basic DDI to the addition of medication administration tracking and various alerts, including pregnancy contraindications, allergy checks, dosage checks, therapeutic duplications, and potentially inappropriate medication (PIM) usage in vulnerable populations at increased risk of ADEs [12,17,29,30,33-35]. One study [30] compared a structured pharmacist medication review supported by a CDSS with a review conducted without CDSS support for older hospitalized patients. The intervention included medication reconciliation and personalized pharmaceutical care (Table 3).

Of the 13 studies, 4 (30.8%) [10,31,32,36] showed that the implementation of automated medication-dispensing systems has been increasingly used to reduce dispensing and administration errors, enhance workflow efficiency, and improve stock inventory tracking. These systems typically incorporated automated individual unit-dose dispensing, which ensured precise medication packaging tailored to patient-specific prescriptions, unlike manual systems where nurses prepare doses manually [31,32,36]. The systems were often integrated with barcode scanning technology, either at the hospital pharmacy or at the patient bedside level, enabling verification of both the medication and the patient’s identity to prevent administration errors [31,32]. Additional functionalities included structured drug storage and controlled access (eg, automated dispensing cabinets [ADCs] and medicine carousel systems with rotating shelves or bins to minimize the time spent searching for medications and medication selection errors) [10,32,36]. One intervention also improved inventory management by providing automated real-time stock monitoring [10]. Furthermore, some systems were linked with electronic medication administration records and other CDSS tools, allowing better coordination between dispensing and prescribing processes [31,32,36].

**Table 3:** Overview of key characteristics of the 13 included studies.

Author	Country	DHT^a^ type	Target population	Sample size	Type of study	Time horizon
Vermeulen et al [12]	The Netherlands	CDSS^b^/CPOE^c^	Patients admitted to the internal medicine, gastroenterology, or geriatric ward	1195	CEA^d^	Admission to discharge
Westbrook et al [33]	Australia	CDSS/CPOE	Patients admitted to the cardiology ward	1202	CEA	15 years
Wu et al [34]	Canada	CDSS/CPOE	Patients admitted to all wards	74,351	CEA	10 years
Avery et al [15]	United Kingdom	IT-based pharmacist outreach	Targeted patients based on conditions and medications in general practices	480,942	CEA	6 months
Berdot et al [10]	France	Automated medication dispending	Patients admitted to all wards	70,421	CBA^e^	1 year
Forrester et al [29]	United States	CDSS/CPOE	Patients in multidisciplinary outpatient clinics	10,080	CEA	5 years
Elliot et al [37]	United Kingdom	IT-based pharmacist outreach	Targeted patients based on conditions and medications in general practices	480,942	CUA^f^	6 months
Gallagher et al [30]	Ireland	CDSS and pharmacist review	Older hospitalized patients	737	CEA	Admission to discharge or 10-day follow-up
Li et al [35]	China	CDSS/CPOE	Inpatients and outpatients	620,000	CBA	6 years
Maviglia et al [36]	United States	Barcode dispensing	Patients admitted to all wards	175,000	CBA	5 years
Nuckols et al [17]	United States	CPOE/CDSS	Patients admitted to all wards	4891^g^	CEA	10 years
Risør et al [31]	Denmark	Automated medication dispensing	Patients admitted to the hematological ward	1336	CEA	6 months
Risør et al [32]	Denmark	Automated medication dispensing	Patients admitted to acute wards	90,000^h^	CEA	6 months

^a^DHT: digital health technology.

^b^CDSS: clinical decision support system.

^c^CPOE: computerized provider order entry.

^d^CEA: cost-effectiveness analysis.

^e^CBA: cost-benefit analysis.

^f^CUA: cost-utility analysis.

^g^Number of acute care hospitals in the United States.

^h^Total number of doses.

A technology-based pharmacist outreach was implemented in 2 (15.4%) studies [15,37], involving pharmacists engaging directly with other health care professionals to target specific high-risk prescribing errors, such as prescribing nonsteroidal anti-inflammatory drugs (NSAIDs) without proton pump inhibitors (PPIs) for patients with a history of peptic ulcers, beta-blockers for patients with asthma, and angiotensin-converting enzyme (ACE) inhibitors or diuretics without proper monitoring of renal function and electrolytes. The interventions involved a CDSS-supported feedback system and pharmacist educational outreach for general practice staff. This intervention model extended beyond simple error reporting by providing pharmacist-intensive support and guidance to other health care professionals.

### Clinical Effectiveness Estimates

The DHT interventions were effective in reducing ADEs, with an average reported effectiveness of 37.12% (range 8.2%-66.5%) across the included studies (Multimedia Appendix 2 and Table 4) [12,17,29,30,34,35]. Medication errors reduced by an average of 54.38% (range 24%-83%) [10,12,15,17,29,31-33,36]. The variability in the effectiveness of DHT in reducing ADEs and medication errors can be influenced by differences in DHT features and the target population. A comprehensive CDSS/CPOE with both prescription entry and administration tracking resulted in greater reduction in ADEs compared to a CDSS/CPOE with only basic alerts [12,34]. Nevertheless, none of the studies provided data on alert compliance tracking by health care professionals. Higher effectiveness was also observed in DHT interventions targeting high-risk populations, such as older and pediatric hospitalized patients. These populations are more prone to ADEs and medication errors resulting from comorbidities, concomitant medications, and differences in physiological characteristics. Consequently, interventions targeting these populations showed greater absolute reductions in ADEs and medication errors compared to interventions targeting lower-risk populations, as the elevated baseline risk provides more room for improvement [29,30].

**Table 4:** Examples of ADEs^a^ and medication errors assessed in the included studies.

Safety outcomes	Examples	Reduction (%), mean (range)
ADEs	Dyspepsia associated with NSAIDs^b^, antiplatelets, and corticosteroids use Severe hypotension resulting from ACE^c^ inhibitors in patients with volume depletion Acute kidney injury caused by NSAIDs prescribed without proper renal monitoring Hyperkalemia due to the concurrent use of ACE inhibitors and potassium-sparing diuretics Bleeding complications caused by an inappropriate dose of anticoagulants Hypoglycemia resulting from insulin overdose Bradycardia from incorrect beta-blocker dispensing Falls and fractures caused by benzodiazepine use in older patients Renal toxicity from aminoglycosides prescribed without dose adjustment for kidney function Hypotension and syncope caused by inappropriate antihypertensive drug combinations Cardiac arrhythmia caused by QT-prolonging drug combinations Severe allergic reactions from failure to recognize and document allergies	37.12 (8.20-66.50)
Medication errors	Incorrect drug dosing due to incorrect weight-based calculation and transcription error Failure to adjust medication doses for patients with renal impairment NSAIDs without PPIs^d^ in patients with ulcer history Beta-blockers prescribed to patients with asthma Dispensing the incorrect medication, strength, or dosage form Errors related to supply failure or expired drugs Monitoring errors, such as patients on ACE inhibitors or diuretics without renal and electrolyte monitoring in the past 15 months, patients on warfarin without a recorded International Normalized Ratio (INR) check in the past 12 weeks, patients on methotrexate without a full blood count or liver function test in the past 3 months	54.38 (24.00-83.00)

^a^ADE: adverse drug event.

^b^NSAID: nonsteroidal anti-inflammatory drug.

^c^ACE: angiotensin-converting enzyme.

^d^PPI: proton pump inhibitor.

### Cost-Effectiveness Estimates

#### CDSS/CPOE Intervention

Implementation of a CDSS/CPOE to replace paper-based prescribing was associated with an average cost ranging from US $25.64 to $81.36 per patient, depending on the complexity and features of the system. The key cost components for a CDSS/CPOE included initial investments in hardware and software, licensing fees, staff training, ongoing system maintenance, and periodic updates to ensure compatibility with clinical workflows (Table 5) [12,17,29,30,33-35]. The implementation cost of a CDSS/CPOE depends on the hospital size, influenced by the need for more extensive hardware infrastructure and software to support a higher number of workstations, higher provider and patient volume, and more comprehensive clinical decision support rules to support various specialties [17]. Most studies evaluating a CDSS/CPOE reported the interventions as cost-effective from perspectives of societal, hospital, and health care systems, with 3 (23.1%) studies [12,17,33] identifying cost-saving results. Only 1 (7.7%) study showed an exceptionally high ICER per ADE prevented without stating a willingness-to-pay (WTP) threshold, highlighting that more data on the effectiveness of CPOE in reducing ADEs are needed [34]. This study was conducted in 2006; therefore, the data on effectiveness may not reflect the most current findings, as presented in more recent studies [12,29,30,33,37]. Although the development of an electronic medical record system with a CDSS requires an initial high investment, 1 (7.7%) study [35] showed that it yields a positive cost-benefit ratio of 1.45, indicating that every US $1 spent generates US $1.45 in benefits, with the ROI achieved within 3 years, driven by a 40% reduction in ADEs as additional treatments and hospitalizations due to ADEs reduced and efficiency improved from transitioning to electronic systems [35].

**Table 5:** Cost components associated with the DHT^a^ interventions included in this review.

Author	DHT type	Currency, year	Cost components	Discount rate (%)
Vermeulen et al [12]	CDSS^b^/CPOE^c^	Euro, 2009	Software, hardware, and maintenance specific to the CDSS/CPOE Costs for personnel involved in setting up and maintaining the system Maintenance and operational costs: ongoing expenses for system updates and operational requirements	Not reported (follow-up period did not exceed 1 year)
Westbrook et al [33]	CDSS/CPOE	AU $, 2012-2013	Software license fees, infrastructure upgrades, and equipment specific to the electronic medication management system Personnel training and configuration Ongoing operating costs: annual software licensing fees, routine system maintenance, and regular training sessions for personnel	5
Wu et al [34]	CDSS/CPOE	US $, 2007	Software and hardware setup System configuration and testing Training sessions for doctors, nurses, and support staff on using the new electronic system Ongoing operational and maintenance costs: software updates and maintenance, licensing fees, and periodic staff training Workload costs (considered in sensitivity analysis)	5
Avery et al [15]	IT-based pharmacist outreach	UK pound sterling, 2012	Report generation Pharmacist training Error management activities, including review of patient medical records, consultations with general practitioners, patient follow-up (eg, patient counselling and medication adjustment, if needed), and training for general practice staff by pharmacists	Not reported (follow-up period did not exceed 1 year)
Berdot et al [10]	Automated medication-dispensing system	Euro, 2015	Purchase of dispensing units, including software and licenses Immobilized drug stock Annual maintenance Labor costs: pharmacy technician wages for ADC^d^-related tasks	Not reported
Forrester et al [29]	CDSS/CPOE	US $, 2010	Hardware, software, and system setup Administrative costs: prescription processing, including chart pulls and queuing Incentives: financial incentives related to meaningful use and pay-for-performance criteria Maintenance costs Personnel costs for implementation, training, and support	3
Elliot et al [37]	IT-based pharmacist outreach	UK pound sterling, 2012	Report generation Pharmacist training Error management activities, including review of patient medical records, consultations with general practitioners, patient follow-up (eg, patient counselling and medication adjustment, if needed0, and training for general practice staff by pharmacists	3.5
Gallagher et al [30]	CDSS and pharmacist review	Euro, 2012	Pharmacist time and training: cost of pharmacists applying structured medication reviews and the CDSS Health care staff review time: physician and nurse time for reviewing care plans Hospital inpatient day: cost of inpatient care per day Software and training costs	Not reported (follow-up period did not exceed 1 year)
Li et al [35]	CDSS/CPOE	US $, 2009	Hardware and software costs Implementation costs: workflow setup, training, and transition Maintenance costs: ongoing system support and utilities	10
Maviglia et al [36]	Barcode-dispensing system	US $, 2009	Planning cost: workflow redesign and stakeholder engagement Software development: linking the barcode system with the existing CPOE system, medication inventory, dose verification tracking, interface design, etc Equipment purchase and infrastructure changes Training cost	3
Nuckols et al [17]	CDSS/CPOE	US $, 2012	Implementation cost: hardware, software, training, and technical support Provider workflow cost	3
Risor et al [31]	Automated medication-dispensing system	Euro, 2017	System integration: development of interfaces between electronic medication administration records, scanners, and the barcode-scanning system Equipment purchase: automated medication-dispensing machine and barcode-scanning devices Operational costs: cost of dose packaging, pharmaceutical services for prescription checks, and additional labor costs Training cost: training for nurses and pharmacy staff on AMS^e^ processes	Not reported (follow-up period did not exceed one year)
Risor et al [32]	Automated medication-dispensing system	Euro, 2018	System implementation: establishment of electronic medication administration records tailored to AMS types Equipment purchase: automated medication-dispensing machines, scanners, and ADCs (complex automated medication system [CAMS] only) Operational costs: maintenance costs, dose bag handling, and additional pharmacy labor for prescription checks (only for patient-specific automated medication system [PSAMS] and CAMS) Training cost: education programs tailored to the AMS complexity used in each ward	Not reported (follow-up period did not exceed one year)

^a^DHT: digital health technology.

^b^CDSS: clinical decision support system.

^c^CPOE: computerized provider order entry.

^d^ADC: automated dispensing cabinet.

^e^AMS: automated medication system.

#### Automated Medication-Dispensing System

Implementation of automated medication-dispensing systems to replace traditional floor stock systems was cost-effective and beneficial, with the ICER ranging from US $0.33 to $62.00 per medication error avoided [10,31,32,36]. Key cost components for an automated medication-dispensing system included purchase of dispensing cabinets or medicine carousel systems with rotating shelves or bins, equipment for the repackaging center, software development, a barcode scanner, labor for restocking and packaging, training expenses, and system integration with the existing CPOE and pharmacy system. A reported net benefit of US $5,379,938.17 was achieved over 5 years, with the ROI attained within 4.25 years after initiation [10,36]. The key drivers of the cost-effectiveness included handling costs and differences in the rates and types of medication errors [31,32,36]. Different types of dispensing errors have varying cost consequences; for example, the administration of an incorrect drug, dosage, or strength can have more severe consequences than procedural or administrative errors, which may potentially but not necessarily result in actual harm [32].

#### IT-Based Pharmacist Outreach

The implementation of a pharmacist-led DHT intervention was cost-effective in reducing clinically significant prescribing and monitoring errors in primary care, with an ICER of US $129.8 per medication error prevented and US $7788 per quality-adjusted life year (QALY) per practice [15,37]. In addition, this intervention not only improved health outcomes (ie, additional QALYs) but also reduced overall costs compared to usual care, indicating its potential for cost savings [37]. Key cost components included pharmacist training, the time spent reviewing patient records and consulting with general practitioners (GPs), report generation from the CDSS, facilitation of practice meetings, patient follow-up, and system integration with the existing IT infrastructure. The number of patients per general practice was a key driver of cost-effectiveness in this intervention. In larger practices, intervention costs (eg, pharmacist training and CDSS report generation) are distributed across a broader patient base. In addition, the targeted nature of this intervention, which addressed specific prescribing errors (ie, NSAIDs without PPIs, beta-blockers for asthma, and monitoring errors) increased the efficiency and impact of pharmacists’ and GPs’ time in practices with higher patient volumes due to economies of scale [15,37,38].

### Methodological Characteristics and Challenges in the Included Studies

Of the 13 studies 6 (46.2%) [10,12,17,31,32,35] used a quasi-experimental design (ie, before-after and interrupted-time-series approaches, with multiple data points tracking ADEs and medication errors before and after DHT initiation). A decision analytic model was used in 3 (23.1%) studies [29,33,35], focusing on short-term, event-specific analysis, while a Markov model was used in 1 (7.7%) study [37]. Randomized controlled trial (RCT)–based evaluation was used in 2 (15.4%) studies [15,30]. Challenges related to methodological aspects of evaluating DHT interventions for medication safety are presented in Table 6. Methodological challenges included short follow-up times, the absence of CDSS alert compliance tracking, a lack of ADE severity classification, and the omission of indirect costs (eg, productivity loss and caregiver time).

**Table 6:** Methodological challenges in DHT^a^ intervention evaluation for medication safety.

DHT type and methodological issues		Description
**CDSS^b^** **/CPOE^c^**		
	Study design	The short-term duration of the study (hospital stay until discharge or 10-day follow-up) did not capture the medium- or long-term impact of the intervention [12,30]. Studies were not able to track whether or how many CDSS alerts were acknowledged and acted upon by health care professionals, limiting accurate assessment of DHT’s effectiveness [12,17,29,30,33-35]. There was no standard care comparator. Before-after designs may not fully account for external factors influencing the observed trend [12,33,35]. The use of a parallel control group for direct comparison could help mitigate confounding effects.
	Cost data	Studies included a societal perspective but lacked data on indirect patient costs (eg, productivity loss, caregiver time) [17].
	Clinical data	There was a lack of detailed ADE^d^ severity classification, which may introduce variability in assessing ADE consequences and the benefits of the intervention [33]. Few studies assessed direct patient-centered outcomes (eg, hospital readmissions, QALY^e^) [17].
**Automated medication-dispensing system**		
	Study design	Studies did not analyze whether identified dispensing errors were tracked or immediately corrected (real-time monitoring), relying on retrospective analysis [10,31,32,36]. Studies were conducted only in hospital settings, limiting generalizability to different settings.
	Cost data	Studies did not explore how saved handling time could be reallocated to other productive tasks (ie, opportunity cost).
	Clinical data	Studies focused on refill errors and urgent deliveries but did not systematically track errors or discrepancies between prescription and dispensing records [10].
**Pharmacist-led IT intervention**		
	Study design	Studies focused on specific high-risk prescribing and monitoring errors but did not directly assess the proportion of errors leading to ADEs [15,37].

^a^DHT: digital health technology.

^b^CDSS: clinical decision support system.

^c^CPOE: computerized provider order entry.

^d^ADE: adverse drug event.

^e^QALY: quality-adjusted life year.

### Quality of Reporting

Based on the quality assessment using the CHEERS checklist, most of the included studies (n=10, 76.92%) [12,15,17,29-33,36,37] were rated as good, and the remaining 3 (23.1%) studies [10,34,35] were rated as moderate. Adherence to the checklist items varied across the section. Most studies did not report any approach to engagement with patients in the study design and results, except for the RCT-based study [30]. The rationale for selecting the model was inadequately reported in over a third of the studies, indicating issues in validating the selected methodology [10,30,31,34,36]. All studies reported how to measure ADEs and medication errors; however, the valuation of these safety outcomes was not reported in some studies [10,12,33]. The components of the outcomes included costs related to additional treatments and hospital stays due to the occurrence of ADEs and medication errors. Although most studies conducted sensitivity analysis to assess the robustness of the findings, only 1 (7.7%) study [32] assessed the heterogeneity of the outcomes based on different types of automated medication-dispensing systems. Most studies only focused on aggregated data without exploring subgroup differences, such as variations within patient populations, hospital wards, or intervention types.

## Discussion

### Principal Findings

This is the first systematic review that assessed the economic evaluations of DHT interventions to improve medication safety outcomes. More than half of the studies (n=7, 53.9%) focused on a CDSS/CPOE, less than a third (n=4, 30.8%) on automated medication-dispensing systems, and the remaining (n=2, 15.4%) on pharmacist-led outreach programs targeting health care professionals. On average, DHT interventions reduced ADEs by 37.12% and medication errors by 54.38%. In 92.3% (12/13) of the included studies, the DHT was either cost-effective or cost beneficial compared to standard care. Despite a significant upfront cost, DHT showed an ROI within 3-4.25 years. Key methodological challenges included short follow-up periods, a lack of ADE severity categorization, the absence of alert compliance tracking, and the omission of indirect costs.

A CDSS/CPOE has been increasingly used to support clinicians in making informed decisions by providing recommendations based on patient data and clinical guidelines [39]. Nevertheless, the effectiveness of a CDSS is often compromised by alert fatigue, which occurs when clinicians override a large number of potentially irrelevant drug safety alerts (eg, drug interactions that are not clinically significant, flagging dosages outside the standard guideline when the prescribed dosage is appropriate for the patient condition) [40-42]. A previous study showed that alert fatigue might be reduced by using a CDSS/CPOE with interactive features, such as incorporating tiered safety alerts with varying priority levels, offering action plans for clinicians (eg, dose reduction), and requiring clinicians to justify overriding an alert [41]. In our review, none of the studies tracked CDSS/CPOE alert compliance, which may influence their effectiveness. A previous study showed drug allergy alerts had the highest compliance [43]. Further DHT evaluation studies should investigate how alert compliance affects the outcomes to ensure optimal utility of DHT.

In addition, tailoring clinical roles within a CDSS can also enhance alert acceptance [44-46]. A pharmacist-mediated CDSS has improved prescriber acceptance by filtering irrelevant advice and providing actionable drug safety recommendations [44]. This approach leverages pharmacists’ expertise related to medication to support the decision-making process and reduce alert fatigue among prescribers [47-49]. Several included studies combined a CDSS with pharmacist-led interventions, such as structured medication review and targeted training for health care professionals for error correction, highlighting the central role of pharmacists in medication safety [15,30,37].

Advancements in CDSSs/CPOE have significantly reduced prescribing errors, but administration errors, such as administering the wrong dose, using the incorrect route of administration, and failing to administer a scheduled dose, still present substantial room for improvement [50]. This review found that a CDSS/CPOE with integrated prescription entry and administration tracking that address prescribing errors, while also monitoring and mitigating administration errors, achieves a greater reduction in ADEs compared to systems with basic features [12,34]. The development and implementation of more comprehensive systems that target all stages of medication management, including administration, are essential to reduce medication errors and ADEs.

Different types of automated medication-dispensing systems were used in the included studies. Several factors need to be considered when selecting an automated medication-dispensing system, including the volume of prescriptions and types of medication the device can handle (eg, oral tablets, liquid medications, injectables), integration with the existing information technology system, and the presence of an error-checking mechanism [51,52]. Almaki et al [53] and Tsao et al [54] showed that integration with existing digital infrastructure is key to supporting a seamless workflow process to ensure all components of medication management (ie, prescribing, dispensing, and inventory control) are interconnected, enhancing efficiency and medication error prevention.

All the included studies focused on hospital-based automated medication-dispensing systems, with limited investigation in outpatient settings [10,31,32,36]. Williams et al [52] demonstrated that automated medication-dispensing systems for chronic medication regimens, such as antiretroviral therapy, enable efficient one-time password (OTP)–based medication collection and reduced waiting times, benefiting high-volume, resource-limited outpatient clinics. Further studies may adopt this system for less complex medication regimens to improve patient satisfaction and reduce the staff burden, enabling health care providers to focus on other critical tasks, such as optimization of drug therapy [54,55].

Targeting medication errors with a substantial clinical impact was a key strategy in the technology-based pharmacist-led outreach [15,37]. As around 1% of medication errors lead to actual harm, prioritizing interventions is essential [24]. The National Coordinating Council for Medication Error Reporting and Prevention (NCC MERP) developed an index to standardize the classification of medication errors based on severity (ie, no error, error-no harm, error-harm, and error-death), which has been adopted in the hospital setting [56,57]. An initiative to standardize the interception of medication errors using this severity classification index has proven effective to prevent clinically significant patient harm and provide substantial cost savings to the health system [58].

Various economic models were used in the included studies (eg, quasi-experimental design, RCT, decision analytic model), indicating the complexity and multifaceted nature of DHT for medication safety. Sculpher et al [59] emphasized the importance of placing an RCT within a broader framework of evidence synthesis and decision analysis in economic evaluation studies to balance internal validity and broader applicability. Quasi-experimental design can be practical for assessing real-world effectiveness and scalability. However, the assessment of confounders that might affect the observed trend and the lack of guidance in conducting economic evaluations alongside quasi-experimental trials necessitate careful interpretation of the findings [60].

### Strengths and Limitations

This study is the first systematic review investigating economic evaluations of DHT interventions to improve medication safety outcomes. We conducted a rigorous literature search with a comprehensive strategy encompassing a wide range of DHT intervention types to provide a thorough review of different strategies to improve medication safety. We also included evaluations on methodological challenges in DHT intervention assessment to inform future research direction.

Nevertheless, our review has several potential limitations. First, the generalizability of findings may be restricted to high-income settings, as most included studies were conducted in such contexts. Second, heterogeneity in health care settings, study designs, and economic evaluation methods may hinder direct comparisons across studies. Despite this, a narrative synthesis was developed to integrate and interpret the findings. Third, the exclusion of non-English studies may have limited the comprehensiveness of the evidence base. Finally, there was a lack of medication error and ADE severity classification among the included studies. Since different medication errors and ADEs have varying cost implications, this variability makes the interpretation of overall cost-effectiveness less straightforward.

### Conclusion

DHT interventions are economically viable to improve medication safety, with substantial reduction in ADEs and medication errors. On average, DHT interventions reduced ADEs by 37.12% and medication errors by 54.38%. In 92.3% of the included studies, DHT was either cost-effective or cost beneficial compared to standard care. Despite a significant upfront cost, DHT showed an ROI within 3-4.25 years. The key drivers of cost-effectiveness include reductions in outcomes, the proportion of errors resulting in ADEs, and implementation costs. Key methodological challenges included short follow-up periods, the absence of compliance tracking, the lack of ADE severity categorization, and the omission of indirect costs. Future studies should prioritize incorporating alert compliance tracking, ADE and medication error severity classification, and the evaluation of indirect costs, thereby increasing clinical benefits and economic viability.

## Appendix

Multimedia Appendix 1 PRISMA (Preferred Reporting Items for Systematic Reviews and Meta-Analysis) checklist.

Multimedia Appendix 2 Clinical and economic outcomes of the included studies.

## Abbreviations

ACE: angiotensin-converting enzyme
ADC: automated dispensing cabinet
ADE: adverse drug event
AMS: automated medication system
CBA: cost-benefit analysis
CBR: cost-benefit ratio
CDSS: clinical decision support system
CEA: cost-effectiveness analysis
CHEERS: Consolidated Health Economic Evaluation Reporting Standards
CMA: cost minimization analysis
CPOE: computerized provider order entry
CUA: cost-utility analysis
DDI: drug-drug interaction
DHT: digital health technology
GP: general practitioner
ICER: incremental cost-effectiveness ratio
MeSH: Medical Subject Headings
mHealth: mobile health
NSAID: nonsteroidal anti-inflammatory drug
PPI: proton pump inhibitor
PRISMA: Preferred Reporting Items for Systematic Reviews and Meta-Analyses
QALY: quality-adjusted life year
RCT: randomized controlled trial
ROI: return on investment
