# Supplementary material for: Digital Health Technology Interventions for Improving Medication Safety: Systematic Review of Economic Evaluations
Source: J Med Internet Res. 2025 Feb 5;27:e65546. doi: 10.2196/65546 (PMC11840376; doi:10.2196/65546)
Supplement: Multimedia Appendix 2 [file jmir_v27i1e65546_app2.docx]

**Multimedia Appendix 2.** Clinical and economic outcomes of the included studies.

| Author | Intervention | Comparator | Effects | Cost (2024 US $) | ICER (2024 US $) | Cost-effective? | Key cost-effectiveness drivers |
| --- | --- | --- | --- | --- | --- | --- | --- |
| Vermeulen et al [1] | CDSS^a^/CPOE^b^ with basic dosage and DDI^c^ alerts | Paper-based prescribing | 40.3% decrease in medication errors  8.2% decrease in ADEs^d^ | 25.64 per patient for CDSS  21.27 per patient for paper based | 6.09 per error prevented  554.84 per ADE prevented | Yes, from the hospital perspective | Implementation cost of the system, reduction in errors |
| Westbrook et al [2] | CDSS/CPOE supporting both prescription entry and administration tracking  Safety alerts: drug allergies, therapeutic duplication, and dose checks | Paper-based prescribing | 71% moderate-to-serious errors | 80.19-81.36 per admission for CDSS  136.85-140.47 per admission for paper based | 58.09 saving per admission | Dominant, from the health system perspective | Reduction in the cost of moderate-to-serious errors |
| Wu et al [3] | CDSS/CPOE supporting both prescription entry and administration tracking  Safety alerts: drug allergies, DDIs, and therapeutic duplication | Paper-based prescribing | 21% reduction in ADEs | 4.83 million | 20,191.45 per ADE prevented | Not reported | Baseline ADE rates and implementation costs, particularly if the physician workload increased |
| Avery et al [4] | CDSS feedback, followed by pharmacist outreach and support to health care professionals, targeting specific medication errors | CDSS feedback with simple educational materials, without direct pharmacist involvement | NSAIDs^e^ without PPIs^f^ for patients with a history of peptic ulcer: 42% reduction  Beta-blockers for patients with asthma: 27% reduction  ACE^g^ inhibitors/diuretics without renal and electrolyte monitoring: 49% reduction | 6 months: 2076.96 per general practice | 129.8 per error prevented | Yes, from the UK National Health Service (NHS) perspective | Number of patients per practice |
| Berdot et al [5] | ADCs^h^ with structured drug storage, controlled access, and automated inventory monitoring | Traditional floor stock storage | 24% reduction in medication errors | 5 years: 905,036.15  After 5 years:  597,125.26 | Not reported  CBA^i^ result: ADCs justify high upfront costs with long-term savings from fewer errors and increased efficiency | — | Decrease in urgent deliveries, fewer errors, staff satisfaction |
| Forrester et al [6] | CDSS/CPOE with basic dosing guidance, checks for duplicate therapies, and pediatric dosing calculations | Paper-based prescribing | 55% reduction in medication errors  66.5% reduction in ADEs | 5470.45 per provider per year | 154.29 per ADE prevented | Dominant, from the medical group perspective | Salary and number of specialty care providers, number and cost of chart pulls, number of prescriptions |
| Elliot et al [7] | CDSS feedback, followed by pharmacist outreach and support to health care professionals, targeting specific medication errors | CDSS feedback with simple educational materials, without direct pharmacist involvement | NSAIDs without PPIs for patients with a history of peptic ulcer: 35% reduction  Beta-blockers for patients with asthma: 17% reduction  ACE inhibitors/diuretics without renal and electrolyte monitoring: 36% reduction | 2202.27 per year | 7788 per QALY^j^ per practice | Dominant, from the UK NHS perspective | GP^k^ practice size and types of prescribing errors |
| Gallagher et al [8] | Structured pharmacist review with CDSS support targeting older hospitalized patients | Pharmacist reviews without CDSS support | 33% reduction in ADEs | 203.2 per patient | 1327.16 saving per patient | Dominant, from the Ireland health service executive (HSE) perspective | Reduction in ADEs, reduction in the length of hospital stay |
| Li et al [9] | Electronic medical record system with a basic CDSS (ie, DDI alerts) | Paper-based medical record system | 40% reduction in ADEs | 688,890.92 | Cost-beneficial  CBR^l^: 1.45  Net benefit over 6 years: 793,556.16  ROI^m^: 3 years since initiation | — | Reduced labor and materials for new medical record creation |
| Maviglia et al [10] | Barcode-assisted dispensing system with repackaging center for unit-dose barcode labeling, error checking, integration with the pharmacy IT system, and carousel storage for accurate retrieval | Conventional dispensing system | 63.16% reduction in medication errors (ie, wrong strength, dosage form, and medication) | 3,453,026.22 | Cost-beneficial  Net benefit over 5 years: 5,379,938.17  ROI: 4.25 years since initiation | — | Cost of errors, proportion of errors leading to ADEs |
| Nuckols et al [11] | CPOE/CDSS with safety alerts, including allergy checks, DDI, dosage checking, therapeutic duplication, and clinical monitoring recommendation. | Paper-based prescribing | 54% reduction in medication errors and ADEs | 25-72 beds: 2,843,481.19  72-141 beds:  7,578,120.17  141-267 beds:  15,432,764.83  276-2,249 beds: 35,335,784.24  Lifespan: 8-20 years | 35335784.24 per QALY | Dominant, from the societal perspective | Reduction in medication errors and the proportion of errors leading to ADEs |
| Risor et al [12] | Automated medication-dispensing system, including:   - Electronic medication administration records - Automated individual unit dose–dispensing system - Bedside barcode scanning of medication and patient wristbands for verification | Manual dispensing system using ward stock supplies. Nurses dispense medication based on prescriptions and deliver them manually to patients without automated tools. | 57% reduction in administration errors  94% reduction in clinical errors  Nonsignificant reduction in procedural errors | 6073.92 for 6 months | 0.33 per administration error avoided  0.47 per procedural error avoided  3.14 per clinical error avoided | Yes, from the hospital perspective | Handling costs, including labor costs associated with managing medications during the administration process and packaging costs |
| Risor et al [13] | Patient-specific automated medication system (PSAMS), including pharmacist verification  Non-patient-specific automated medication system (NPSAMS), without pharmacist verification  Complex automated medication system (CAMS), including pharmacist verification and integrated medications stored in an ADC instead of ward rooms | Manual dispensing system using ward stock supplies. Nurses dispense medication based on prescriptions and deliver them manually to patients without automated tools. | PSAMS:   - 83% reduction in administration errors - 90% reduction in procedural errors - 67% reduction in clinical errors   NPSAMS:   - 47% reduction in administration errors - 40% reduction in procedural errors - 67% reduction in clinical errors   CAMS:   - 67% reduction in administration errors - 60% reduction in procedural errors - 33% reduction in clinical errors | PSAMS: 51,416.06  NPSAMS: 5038.76  CAMS: 18,792.72 | PSAMS: 3.86 per clinical error avoided  NPSAMS: 4.18 per clinical error avoided  CAMS: 62 per clinical error avoided | PSAM most cost-effective from the hospital perspective | Reduction in different types of medication errors |

^a^CDSS: clinical decision support system.

^b^CPOE: computerized provider order entry.

^c^DDI: drug-drug interaction.

^d^ADE: adverse drug event.

^e^NSAID: nonsteroidal anti-inflammatory drug.

^f^PPI: proton pump inhibitor.

^g^ACE: angiotensin-converting enzyme.

^h^ADC: automated dispensing cabinet.

^i^CBA: cost-benefit analysis.

^j^QALY: quality-adjusted life year.

^k^GP: general practitioner.

^l^CBR: cost-benefit ratio.

^m^ROI: return on investment.

## References

1. Vermeulen K, van Doormaal J, Zaal R, Mol P, Lenderink A, Haaijer-Ruskamp F, Kosterink J, van den Bemt P. Cost-effectiveness of an electronic medication ordering system (CPOE/CDSS) in hospitalized patients. *Int J Med Inform* 2014 Aug; 83(8):572-580.
2. Westbrook J, Gospodarevskaya E, Li L, Richardson K, Roffe D, Heywood M, Day R, Graves N. Cost-effectiveness analysis of a hospital electronic medication management system. *J Am Med Inform Assoc* 2015 Jul; 22(4):784-793.
3. Wu RC, Laporte A, Ungar WJ. Cost-effectiveness of an electronic medication ordering and administration system in reducing adverse drug events. *J Eval Clin Pract* 2007 Jun 18; 13(3):440-448.
4. Avery AJ, Rodgers S, Cantrill JA, Armstrong S, Cresswell K, Eden M, Elliott RA, Howard R, Kendrick D, Morris CJ, Prescott RJ, Swanwick G, Franklin M, Putman K, Boyd M, Sheikh A. A pharmacist-led information technology intervention for medication errors (PINCER): a multicentre, cluster randomised, controlled trial and cost-effectiveness analysis. *Lancet* 2012 Apr; 379(9823):1310-1319.
5. Berdot S, Blanc C, Chevalier D, Bezie Y, Lê LMM, Sabatier B. Impact of drug storage systems: a quasi-experimental study with and without an automated-drug dispensing cabinet. *Int J Qual Health Care* 2019 Apr 01; 31(3):225-230.
6. Forrester SH, Hepp Z, Roth JA, Wirtz HS, Devine EB. Cost-effectiveness of a computerized provider order entry system in improving medication safety ambulatory care. *Value Health* 2014 Jun; 17(4):340-349.
7. Elliott RA, Putman KD, Franklin M, Annemans L, Verhaeghe N, Eden M, Hayre J, Rodgers S, Sheikh A, Avery AJ. Cost effectiveness of a pharmacist-led information technology intervention for reducing rates of clinically important errors in medicines management in general practices (PINCER). *Pharmacoeconomics* 2014 Jun 18; 32(6):573-590.
8. Gallagher J, O'Sullivan D, McCarthy S, Gillespie P, Woods N, O'Mahony D, Byrne S. Structured pharmacist review of medication in older hospitalised patients: a cost-effectiveness analysis. *Drugs Aging* 2016 Apr 9; 33(4):285-294.
9. Li K, Naganawa S, Wang K, Li P, Kato K, Li X, Zhang J, Yamauchi K. Study of the cost-benefit analysis of electronic medical record systems in general hospital in China. *J Med Syst* 2012 Oct 3; 36(5):3283-3291.
10. Maviglia SM. Cost-benefit analysis of a hospital pharmacy bar code solution. *Arch Intern Med* 2007 Apr 23; 167(8):788.
11. Nuckols TK, Asch SM, Patel V, Keeler E, Anderson L, Buntin MB, Escarce JJ. Implementing computerized provider order entry in acute care hospitals in the United States could generate substantial savings to society. *Jt Comm J Qual Patient Saf* 2015 Aug; 41(8):341-350.
12. Risør BW, Lisby M, Sørensen Jan. Cost-effectiveness analysis of an automated medication system implemented in a Danish hospital setting. *Value Health* 2017 Jul; 20(7):886-893.
13. Risør BW, Lisby M, Sørensen J. Comparative cost-effectiveness analysis of three different automated medication systems implemented in a Danish hospital setting. *Appl Health Econ Health Policy* 2018 Feb 8; 16(1):91-106.
